# Fear, freedom and political culture during COVID-19

**DOI:** 10.1007/s40592-022-00157-5

**Published:** 2022-06-15

**Authors:** Tim Soutphommasane, Marc Stears

**Affiliations:** 1 The University of Sydney, Sydney, Australia; 2 University College London, London, England

## Abstract

Australia’s experience of the COVID-19 pandemic has been widely perceived to have been a successful one, based on the relatively few number of lives lost to the virus compared to the rest of the world. There remain, nonetheless, serious ethical challenges at the heart of the Australian response to COVID-19. The broadly positive outcomes of Australia’s pandemic response mask more troubling developments within its political culture, and the costs it has imposed on its society. This article examines two concerns in particular: the normalisation of fear and emergency through the language and policy responses adopted by governments, and the significant diminution of individual freedoms and human rights.

*

As the world has grappled with the pandemic, the experiences of just a small handful of countries are widely perceived to have bucked the trend of mass infections, hospitalisations and deaths. Australia is one of those countries.^1^ For many Australians, if not most, the experience of COVID-19 has been relatively fortunate. Two years into the pandemic, the number of lives lost to the virus have been few compared to the rest of the world. The swift and near absolute closure of Australia’s international and domestic borders, along with Australian citizens’ willingness to support government policies – including lockdowns and effective tracking, tracing and isolation measures – have prevented the virus from sweeping through the country with the same devastation as it has elsewhere.

Australian governments also acted quickly on the economic front. Early on in the pandemic, then Prime Minister Scott Morrison declared there was a twin crisis: a health one and an economic one. Although he was a little slower off the mark than some northern European countries, his government introduced one of the world’s largest stimulus packages to cushion the economic shock of Australian society locking down.^2^ There was no way, of course, that any government could prevent the Australian economy – untouched by a downturn for three decades – heading into recession. But government intervention prevented the unemployment rate from jumping into double figures. Probably as a result, the intense political dysfunction that has gripped many countries, epitomised by dangerous forms of nationalist populism and a rejection of scientific expertise, has been absent. The Australian system has remained resolutely centrist during the entirely of the pandemic.^3^

There have been, nonetheless, serious ethical challenges at the heart of the Australian response to COVID-19. Indeed, we believe that the broadly positive outcomes mask more troubling developments, both specifically within the response to the pandemic and the broader political culture. In this article, we reflect on the cost that Australia’s COVID-19 response has imposed on its society.

There have been prominent critics of the overall Australian approach to COVID-19 – if not the country itself – right from the outset. The Italian philosopher Giorgio Agamben warned that the willingness of governments to resort to lockdowns and citizens’ compliance with those restrictions, demonstrates that “society no longer believes in anything except *bare* life.” Agamben insisted that the closing down of large sections of the economy, the abandonment of everyday social interactions and the prohibitions on even members of extended families mingling together, displayed a culture now “willing to sacrifice practically everything – normal living conditions, social relations, work, and even friendships, affections, and their religious and political beliefs – when confronted with the danger of getting sick”. Looking beyond the immediate crisis of the pandemic and into the future, Agamben asked, “What happens to human relationships in a country that becomes accustomed to living this way?”^4^

The Australian experience may help to provide an answer. Australia was, along with New Zealand, very rare among democracies in assiduously prioritising “bare life” over all other concerns at almost all stages of the pandemic from March 2020 to November 2021. Unlike most democracies, which partially restricted inward and outward travel during this period, Australia firmly closed its international and most of its domestic state borders too. It allowed inward travel only from citizens and permanent residents on an increasingly restricted quota scheme, and only with two weeks of hotel quarantine. While a small class of visa holders enjoying special “economic exemptions” were also permitted to enter, more than 30,000 Australian citizens were left stranded overseas. At the same time, citizens and permanent residents had to request special permission to leave the country, permission which was more often denied than accepted. On top of this, Australian states regularly deployed of lockdowns, in pursuit of a “zero COVID” policy, where almost any instances of infection were treated as standing in need of an urgent and full-throated response. This strategy led to its second largest city, Melbourne, living through what some describe as the largest number of lockdown days of any major city in the world, with 277 days spent under public health orders that involved restrictions on people leaving home.

There are at least two major ethical concerns raised by this particular Australian response to the pandemic. First, governments have normalised fear and emergency through their language and policy responses – with a special emphasis on the use of police and the military in delivering what are nominally described as “public health” interventions. Second, this response has seen a significant diminution in individual freedoms, which have been comparatively pronounced in Australia because of its lack of constitutionally protected rights and freedoms, not to mention the absence of vibrant political debate about the place of liberties in pandemic responses seen elsewhere.^5^ What is especially striking in these developments has been how limited dissent has been, particularly among those sympathetic to human rights, social democracy and progressive liberalism. Erstwhile reliable stalwarts of rights and freedoms have been noticeable quiet in speaking out against COVID-19’s effects on Australia’s liberal democratic culture. As we explain, this may reflect a certain ideological character to public understanding of the pandemic: the political conflicts revealed by COVID-19 seem to have reinforced divisions and discourses associated with a so-called “culture war” that has come to define contemporary Australian politics.

*

When COVID-19 arrived in Australia in March 2020, the threat was clearly defined in the public mind. As much as it was a matter of public health, it was a threat to Australia’s security and activated a political response to close national borders. It was no accident that, when the federal Parliament sat for the first time after the pandemic hit, the then Prime Minister spoke of the government’s response as being about “defending and protecting Australia’s national sovereignty”.^6^ COVID-19 was described consistently as if it were an enemy combatant in war. Following a resurgence of the virus in Victoria in July 2020, various media reports in New South Wales and Queensland referred to incursions from “Mexicans” from the south, and even to the “Melbourne virus”.^7^ Such rhetoric reinforced an early, popular racialised undercurrent about COVID-19 being a “Chinese virus” (as Donald Trump and many others have called it).^8^ At the time of writing, a number of states were only just beginning to open their borders to Australian travelers from elsewhere; Western Australia’s borders were closed to international visitors until March 2022, with a very few number of exceptions.

Such settings highlight the normalisation of emergency. Australia defaulted to a position of “Fortress Australia” during 2020-21, and for much of that period to the pursuit of a “zero Covid” strategy. Government policy aimed to keep the number of COVID-19 infections at zero, essentially by sealing the borders and keeping the rest of the world out. This was supported by the mindset of an island nation convinced that it has it within its power to keep external threats at bay – a distinctive form of protectionism that was historically directed at restricting immigration from Asia and non-European countries.^9^ Many Australians have been comfortable to have their international borders effectively closed for most of the pandemic so far. A country that has been an open, globally engaged society shifted seamlessly into becoming a closed nation fearful of outsiders, with overseas arrivals regarded as threats to national biosecurity and sovereignty.

The normalisation has gone beyond closed borders. The package of Fortress Australia has also included a pronounced reliance on lockdowns and restrictions, justified as necessary until enough of the population was fully vaccinated. For the most part, Australians have accepted this as part of the reality of living through a global pandemic. Yet to some degree, it has reflected a belief that Australian exceptionalism – its unique ability to rely on closed borders, born of its status as a country without any contiguous borders with another country – could even substitute for vaccination, or at least come to assume more primary importance over time. While other countries directed their attention to approving, procuring and then distributing vaccines at speed, Australia decided, in the words of its former prime minister, that it “isn’t a race”. Some prominent public health experts told Australian citizens in broadcast interviews that, while other countries were using emergency procedures to check the efficacy and safety of vaccines, Australia could afford to take its time and avoid the risks of a rushed vaccine rollout.^10^

That acceptance, however, arguably crept into something more unnerving. A temporary reliance on emergency measures has arguably felt in danger of becoming more permanent – more normal. This was demonstrated in the experience of Australia’s two largest cities, where there has been a militarisation of the pandemic response and an over-policing of our most vulnerable communities. In Sydney, the year 2021 was seen truly a tale of two cities. As a result of an outbreak of the Delta variant of COVID-19 penetrating the western and southwest suburbs of Sydney in June-July, the New South Wales (NSW) state government responded with public health orders involving restrictions on people’s movements. This followed calls by some epidemiologists for military and police to be called upon to enforce the lockdown. According to Tony Blakely, a prominent epidemiological expert based in Melbourne, the NSW government was faced with the options of either “letting the virus rip”, instituting a partial lockdown leaving many retail stores open, or a more extreme lockdown:

The third option is the only one. It’s very hard to say those words: “We’re going into a hard lockdown, we’ve called in the military, the police and we are going to police it.” It’s not a pretty look but if you want it to be over and done with as quickly as possible that, most unfortunately, is what needs to happen.^11^

Shortly after such calls, this was precisely what happened. The NSW government introduced new public health orders placing restrictions on gathering and movement in Greater Sydney, including directions for “affected persons” to stay at home.^12^ It also requested assistance from the Commonwealth Government for the use of Australian Defence Force personnel to help ensure compliance, working alongside NSW police. However, the public health orders underpinning this targeted “local government areas of concern” in western and southwest Sydney – with those in more affluent, coastal parts of the city subjected to less stringent requirements. People in highly multicultural local government areas such as Fairfield, Liverpool and Canterbury-Bankstown had to contend with a military presence on their streets, no outdoors recreation and exercise for only one hour a day. There were curfews in place, despite the NSW government acknowledging there were no solid public health evidence that they impacted on the spread of the virus. These restrictions were not in place for people in more affluent local government areas in Sydney’s northern beaches, eastern suburbs and the inner-west.^13^ Meanwhile in Melbourne, the city not only became subject to some of the longest running lockdowns in the world but also to the most restrictive and, in the view of some critics at least, the most arbitrary. During these lockdowns, even late into 2021, the Victorian government enforced a nightly curfew and closed public playgrounds, without offering any evidence that they were a risk to public health.^14^ And much like in Sydney, some of the measures were targeted at parts of the city that are most socially and economically disadvantaged. In July 2020 the Victorian government issued directions under the *Public Health and Wellbeing Act* to detain residents of public housing towers in North Melbourne and Flemington for an “initial detention period” of 14 days, as the state entered a “Stage 4” lockdown to tackle an emerging COVID-19 outbreak (which involved not merely stay at home orders, but also limits on permitted outdoor exercise, limits on the distance people could travel to obtain essential goods, a curfew and mandatory mask wearing for all people aged over 12).^15^ Unlike their neighbours in private housing on the streets adjoining them, the residents of these towers, many with histories of trauma and most experiencing the stress of ongoing financial hardship, were subjected to a lockdown so harsh it made international news as one of the world’s strictest COVID-19 lockdowns without warning.^16^ Residents of one tower had to wait more than one week before being permitted to go outside, under police supervision, for fresh air; many residents across the towers in question were left without food and medicine. In a formal investigation, the Victorian Ombudsman found that the snap lockdown constituted a serious human rights violation, unnecessarily subjecting people to detention, anguish, confusion, and in the worst cases re-traumatisation.^17^

Culturally, though, the national discourse has been marked by a widespread acceptance of the language and policy of emergency. The Australian public and media has focused continuously on COVID-19 case numbers, to a large extent ignoring other features of the pandemic or its response. This has reflected the deep and profound public influence of the “zero Covid” approach that defined the national and state responses. At the first signs of an outbreak, there have been quick public calls for lockdowns by prominent media celebrities and news commentators (the harder, the better), followed by the relatively swift introduction of restrictions by state authorities. People have generally accepted lockdowns as a necessary instrument of public health, despite pandemic plans prior to 2020 emphasising the importance of using the least restrictive alternatives and despite the origins of “lockdown” as an instrument of prison management. People also widely accepted that inter-state trips – even inter-city trips within states – could be immediately disrupted by snap public health orders restricting movements, including compulsory quarantining for up to two weeks in government monitored facilities. There was very limited public opposition to the process of signing in with QR codes for the purposes of contact tracing, even though this was a requirement not just in sensitive settings but also in various outdoor settings involving small risks of infection. In short, a sense of apprehension and emergency have prevailed to the point of being banally familiar.^18^

*

This willingness to accept the emergency measures, reflects how, for many Australians in the public square, there has been strong acceptance of a trade off between liberty and safety. The absence of public opposition has just been the necessary, and reasonable, price to pay during a global pandemic. If there has been a diminution of personal liberties, it has only been temporary, the argument goes. Once the pandemic passes, we will be able to revert to the old normal.

In our view, however, there is strong reason to believe that the cumulative effect of the pandemic – the lockdowns and the policy responses – has had a troublingly corrosive effect on Australia’s political culture, including on public understanding of the place of core freedoms in Australia’s democratic practices. There has been the continual expansion of executive power. Almost uniquely in the democratic world, for some time the Australian federal Parliament did not meet with its usual frequency and was extraordinarily slow to adjust to the exigencies of remote working. In the first phases of the pandemic, while many workplaces in the country resorted to conducting their business through Zoom and online calls, and other Parliaments around the world have followed suit, federal legislators waited for nearly six months from the pandemic’s arrival to avail themselves of sitting remotely with the benefit of technology. Similar patterns have played out in other parts of the country. The New South Wales Parliament suspended sittings in June 2021 and did not resume its business until October 2021.^19^

Physical distancing and public health concerns also made public gatherings difficult, even though the infection risk posed by outside activities of this sort was widely considered to be very low. The enforcement of public health orders in various jurisdictions have explicitly prevented significant gatherings from taking place. Moreover, public demonstrations or protests have been treated by various quarters as presumptively seditious. When Black Lives Matters protests took place in mid-2020, they were condemned by News Corp newspapers and some conservative politicians for contributing to rising infection numbers – despite the lack of any evidence, and the obvious time lag between the protests in early June 2020 and the surge in cases in July 2020.

There is in all this a strong danger that the political culture is transforming into what some political scientists have described as an “allegiant” culture.^20^ This is a political culture characterised by an emphasis on order and security, deference to authority, limited democratic protest and compliance with institutions. It stands in opposition to an “assertive” culture, which is characterised by civic participation, direct action and skepticism of authority. In some respects, we are possibly seeing a consolidation of a nation of “quiet Australians”.^21^ Before and after the 2019 federal election, the then Prime Minister Scott Morrison spoke approvingly of these compliant compatriots: hard-working people in the suburbs who neither campaign in the streets nor follow the political news every day and are happy for politics to happen without them.^22^ Indeed, for a nation that has been subjected to such significant restrictions on individual freedoms, Australian society has been relatively muted in its dissent. Compared with other liberal democracies, where there have been significant, widespread protests against lockdowns for their effects on liberty, the Australian public has been strikingly compliant. Where they have taken place, public protests appear to have been dominated by fringe political movements and actors, and regarded by most to sit outside the respectable mainstream.^23^

There are several pragmatic reasons for this public acceptance of the diminution of freedom. The slow pace of the vaccine rollout in the first half of 2021 led many to believe that Australia had no reasonable choice other than to err on the side of caution – that restrictions were necessary because the country’s vaccination rates were not sufficiently high to warrant an opening up in the face of the Delta variant. Political factors have also been at play. Burnt by its defeat in the 2019 federal election, and by the potential impact of the virus at state-level, Labor has rarely openly challenged this framing. If anything, Labor premiers in Victoria, Queensland and Western Australia have been among the most enthusiastic proponents of a “zero Covid” approach right up to late 2021 (with West Australian premier Mark McGowan a prominent supporter of the position even into 2022). At the federal level, Labor leader Anthony Albanese critiqued the Coalition government for failing to build dedicated quarantine facilities across the country. For the most part, while in opposition, Labor adopted a cautious view about Australia’s reopening, positioning itself as a critic of the federal government’s slow rollout of the vaccine and its failure to build quarantine facilities.^24^ In effect, this has reinforced the normalised sense of fear and emergency within public debate.

Then there has been the role played by many epidemiologists and health experts in supporting a stance of “Fortress Australia”. This has been reinforced by political leaders justifying their decisions at every turn as being “backed by the medical advice”, or as reflecting them “listening to the science”. Understandably, in the midst of a pandemic, the voices of health and medical experts – particularly those in infectious diseases and epidemiology – have grown influential, if not decisive, in many policy debates. Yet it may also be the case that such influence has served to narrow public understanding about the pandemic. In particular, COVID-19 has been viewed as presenting a public health crisis of a certain kind, namely, one measured in terms of the numbers of deaths and infections. This has been the definitive measure, for many, of the success or failure of Australia’s policy responses. To return to Agamben, society during a public health crisis of this kind has come to resemble one which “no longer believes in anything except *bare* life”.

It is a point that even some epidemiologists appear to concede. As observed by one prominent supporter of the “zero COVID” strategy, epidemiologist Mary-Louise McLaws, reflecting on her role in public debate:

When I’m asked for opinions in Australia, I have been criticised that I’m not considering the economy or mental health … But I try to remind the listeners or the readers that that’s not part of an epidemiologist’s responsibility – that’s leadership. So you focus on one thing only, and that is your understanding of outbreak and pandemic management.^25^

This represents a very particular framing of scientific responsibility, one which can act to foreclose an understanding of the pandemic as implicating a broader *societal* challenge.^26^ While some epidemiologists or medical experts may see that as raising questions beyond their scientific remit, it seems too convenient to assert that it is beyond their professional responsibility to even consider them – not least when they are actively intervening in public policy debates, and advocating for particular positions for government to adopt. The net effect of this, though, may have been to harden public views about the nature of COVID-19’s challenges and to validate a sense that significant sacrifices of freedom are immune from robust questioning given they are backed “by the science” or “the medical advice”. Only belatedly are many beginning to understand that the voices needed within public discussion of the pandemic must include more than just epidemiologists, but also mental health experts, economists, sociologists, psychologists, political scientists and philosophers. And that reasonable people should be able to question policy positions justified by invocations of “the science” or “medical advice”, without having to feel as though they are sanctioning anti-vaxx and conspiracy theory madness.^27^

*

All of this, of course, began to shift as first Delta and then the Omicron variants demonstrated just how unsustainable “zero COVID” was as a strategy. We now know, if there had been any doubt, that the virus causing COVID-19 will not disappear from the world and that it will continue to spread with unnerving ease even with dramatic restrictions. As the majority of the world’s scientists agree, the virus will become endemic and freely circulate. It is as much of a fantasy to think we can eliminate the virus, as it is to think that human beings have not caused our climate to change. A fully and effectively vaccinated population will not mean that the virus will disappear, though it will hopefully provide a reasonable protection against the worst of it.^28^

What is more certain is that the pandemic reveals we remain in dangerous territory for liberal democratic political culture. It was not that long ago that progressives could be counted to be the guardians of human rights and multiculturalism in Australia. It was not that long ago that it was a mark of political allegiance that progressives rejected the cruelty of Australia’s asylum seeker policies and racist dog-whistling against minorities. Yet today, many progressive activists, commentators and policymakers have supported the maintenance of a “Fortress Australia” stance that willfully overlooked its detrimental effect on the broader ethical health of our society.^29^ They remained largely mute to the restriction of liberties in Melbourne and Sydney, restrictions that have on multiple occasions targeted or disproportionately affected migrant and disadvantaged communities in both cities.

This reflects one political effect of COVID-19: the increasing polarisation of Australian public debate on questions of “freedom”. Criticisms of government restrictions of liberties, couched in the language of “freedom” and human rights, have been routinely derided as libertarian excess. The framing of debates about COVID-19 management have seen arguments in favour of “living with COVID” being equated with “letting the virus rip”.^30^ Those advocating for the re-opening of Australian borders or society are suspected of being in the service of free-market fundamentalism or a twenty-first century social Darwinism, which revels in heightening the fears of those most vulnerable to COVID-19. This is plausibly what happens in a political culture that has come to be so profoundly shaped by culture war: everything becomes a proxy for that culture war.^31^

The result of the May 2022 federal election may yet signal that culture war politics could subside. Yet the pandemic, at least for some time, served to reinforce its dominance, in the process corroding Australia's democracy. Inequalities have widened, narrow-minded protectionism has infected Australian political culture and there has been a worrying erosion of rights and liberties. As Agamben reminded us right at the start, bare life matters, of course, but it is not all that does. There is much to rebuild.

## Footnotes

1 See Bromfield, N., & McConnell, A. 2021. Two Routes to Precarious Success: Australia, New Zealand, COVID-19 and the Politics of Crisis Governance. *International Review of Administrative Sciences*, 87 (3), 518–535. 10.1177/0020852320972465.

2 See Wilkins, P., Gilchrist, D., and J. Phillimore. 2021. Independent Review of Emergency Economic Stimulus Measures: Global Financial Crisis and COVID-19. *Australian Journal of Public Administration* 80 (1), 12–28: 10.1111/1467-8500.12437.

3 For contemporary commentaries, see https://www.lowyinstitute.org/the-interpreter/pandemic-democracy and https://www.sydney.edu.au/content/dam/corporate/documents/sydney-policy-lab/democracy-policy-paper.pdf.

4 See https://medium.com/@ddean3000/clarifications-giorgio-agamben-3f97dc7ed67c)

5 See Allen, D. 2022. *Democracy in the Time of Coronavirus*. Chicago: University of Chicago Press.

6 https://www.pm.gov.au/media/ministerial-statement-australian-parliament-house-act-080420.

7 https://www.traveller.com.au/australia-state-border-closures-seven-of-the-strangest-things-in-our-100-years-of-borders-h1p8cm.

8 See https://theconversation.com/donald-trumps-chinese-virus-the-politics-of-naming-136796.

9 For historical and cultural accounts about this mindset, see Lake, M. and H. Reynolds. 2008. *Drawing the Global Colour Line: White Men’s Countries and the Question of Racial Equality*. Melbourne. Melbourne University Press; Hage, G. 1998. *White Nation: Fantasies of White Supremacy in a Multicultural Society*. London. Pluto Press.

10 See https://www.newstatesman.com/politics/2021/07/how-failure-covid-19-has-exposed-dangerous-delusion-fortress-australia.

11 https://www.news.com.au/national/nsw-act/harsher-lockdown-the-only-real-option-left-to-save-sydney-from-covid19/news-story/2b2c5fcdd80460c907fa023c52d618a2.

12 See *Public Health (COVID-19 Temporary Movement and Gathering Restrictions) Order 2021*. To provide some context regarding the proportionality of the Sydney lockdown during this outbreak of the Delta variant, the numbers of new weekly infections in New South Wales peaked at 9,792 in the week ending 11 September 2021 (there were 1,112 new hospitalisations in that equivalent week), in a population of 8.1 million, see https://www.health.nsw.gov.au/Infectious/covid-19/Documents/covid-19-surveillance-report-20210924.pdf.

13 See https://www.sbs.com.au/news/tale-of-two-cities-frustration-over-crowds-at-sydney-beaches/ff6f32db-daac-4389-ac5a-bd94cc34eaf7.

14 https://www.abc.net.au/religion/closed-playgrounds-rebecca-tullock-and-holly-lawford-smith/13522062.

15 For an outline of Stage 4 restrictions in Melbourne and how they compared with other restrictions enforced in Australia, see S. Goldfeld, E. O’Connor, V. Sung, G. Roberts, M. Wake, S. West, and H. Hiscock. 2022. “Potential indirect impacts of the COVID‐19 pandemic on children: a narrative review using a community child health lens”, *Medical Journal of Australia*: 10.5694/mja2.51368.

16 See the discussion in Riboldi, M., Fennis, L., and M. Stears. 2021. *Nurturing Links Across Civil Society: Lessons from Australia’s For-Purpose Sector’s Response to COVID-19*. Sydney Policy Lab. Sydney.

17 Office of the Victorian Ombudsman. 2020. *Investigation into the Detention and Treatment of Public Housing Residents Arising from a COVID-19 ‘Hard Lockdown’ in July 2020.* Parliamentary Report, Victorian Ombudsman.

18 Indeed, a whole organisation of prominent scientists and others was founded to propound these views. See https://theconversation.com/vital-signs-introducing-ozsage-a-source-of-practical-expert-advice-for-how-to-reopen-australia-from-covid-safely-166943.

19 See https://apo.org.au/sites/default/files/resource-files/2021-07/apo-nid313316.pdf.

20 Evans, M., Halupka, M. and G. Stoker. 2018. *How Australians Imagine Their Democracy: The “Power of Us”*. Institute for Governance & Policy Analysis University of Canberra. Canberra.

21 See https://www.abc.net.au/7.30/who-are-scott-morrisons-quiet-australians/11706370.

22 See Gauja, A., Sawer, M., and M. Simms. 2021. *Morrison’s Miracle: The 2019 Australian Federal Election*. Australia National University Press. Canberra. 10.22459/MM.2020.

23 See Bromfield, N. 2021. Governance of the COVID-19 Crisis in Australia. *Australian Politics and Policy: Senior Edition*. Sydney University Press. Sydney: 645–662. DOI:10.30722/sup.9781743328415.

24 See https://thewest.com.au/news/coronavirus/premier-mark-mcgowan-reveals-was-hard-border-may-stay-closed-until-april-2022-ng-b881998370z and https://www.theaustralian.com.au/news/scott-morrison-had-two-jobs-in-the-pandemic-and-failed-on-both/video/3153a0156d76c1770fad624c8fbace1f.

25 https://www.theguardian.com/world/2021/jul/04/i-love-you-australian-epidemiologists-grapple-with-newfound-covid-fame.

26 See Bor, A., Jorgensen, F. J. and M. Petersen. 2021. “The COVID-19 Pandemic Eroded System Support but Not Social Solidarity”. Preprint. 10.31234/osf/io/qjmct.

27 See the discussions in Niker, F. and A. Bhattacharya. 2021. *Political Philosophy in a Pandemic: Routes to a More Just Future*. Bloomsbury. London.

28 For contemporary understandings at the time of writing, see https://www.who.int/news/item/28-11-2021-update-on-omicron.

29 For an earlier statement of our view, see https://www.abc.net.au/religion/tim-soutphommasane-and-marc-stears-covid-why-are-progressives-s/13521952.

30 For more on this argument, see https://www.theatlantic.com/ideas/archive/2021/09/pandemic-australia-still-liberal-democracy/619940/.

31 This is, of course, part of a broader phenomenon. See Allen, D. 2021. *Democracy in the Time of Coronavirus*. Chicago. Chicago University Press and https://democracyjournal.org/magazine/62-special-issue/democracy-in-a-time-of-coronavirus/.

